# The role of probiotics in nosocomial infections

**DOI:** 10.1186/1824-7288-40-S2-A16

**Published:** 2014-10-09

**Authors:** Gian Vincenzo Zuccotti, Fabio Meneghin

**Affiliations:** 1 Department of Pediatrics, University of Milan, Luigi Sacco Hospital, Milan 20157, Italy

## Background

Nosocomial infections are among the leading causes of mortality and morbidity especially in neonatal intensive care unit (NICU) [1]. The intestinal microbiota of the gut is nowadays considered to play an important functional role in the host’s health through nutritional, physiological and immunological processes. For these reasons, probiotics may exert actions of prevention and therapy of infectious diseases.

## Results

The mechanisms of action of probiotics are strain specific but can be summarized mainly in three areas: changes of gut ecology, modulation of gut mucosal barrier and regulation of the immune response through interaction with gut-associated immune system [2]. Several studies regarding the supplementation of probiotics in nosocomial infections have been conducted mainly in adult population. Among pediatric studies major findings have been observed in treatment of acute gastroenteritis, primarily caused by Rotavirus [3,4], and in the prevention of antibiotic associated diarrhea (AAD) [5]. Supplementation with probiotics has proven useful even in the treatment of Clostridium difficile disease (CDD), the most common pathogen involved in AAD [6]. Data from meta-analysis and cochrane review on the prevention of necrotizing enterocolitis (NEC) show an overall benefit of probiotic supplementation [7]. The limitations of the above cited studies are mainly related to heterogeneity in terms of strain, dosage and duration of treatment and the lack of studies on extremely low birth weight preterm infants. Data on nosocomial pneumonia and ventilator-associated pneumonia in neonatal and pediatric age is scanty. In a large randomized, double-blind placebo controlled study, Hojsak et al demonstrated that supplementation with Lactobacillus GG significantly decreased the risk of nosocomial respiratory tract infections [8]. On the other hand, the data from adult studies have been conflicting, with a tendency towards the demonstration of probiotic efficacy in reducing the incidence of ventilator-associated pneumonia [9]. Meticillin-resistant *Staphylococcus aureus* is a multidrug-resistant nosocomial pathogen; a recent review of literature [10] showed that many probiotic strains inhibit MRSA growth in vitro. Furthermore, this review describes that there is little published clinical data on the use of probiotics in prophylaxis or treatment of MRSA-mediated infections.

## Conclusions

Due to the significant heterogeneity between the studies in literature it is not possible to draw consistent conclusions on extensive use of probiotics in prevention and treatment of nosocomial infections, except for acute gastroenteritis, AAD, CDD and NEC.
